# Is Contrast-Enhanced Ultrasonography a New, Reliable Tool for Early-Graft-versus-Host Disease Diagnosis?

**DOI:** 10.3390/jcm13206065

**Published:** 2024-10-11

**Authors:** Lavinia-Eugenia Lipan, Simona Ioanitescu, Alexandra-Oana Enache, Adrian Saftoiu, Alina Daniela Tanase

**Affiliations:** 1 University of Pharmacy and Medicine “Carol Davila”, 020021 Bucharest, Romania; simona.ioanitescu@gmail.com (S.I.); oanaaaenache@gmail.com (A.-O.E.); adrian.saftoiu@umfcd.ro (A.S.); alinadanielatanase@yahoo.com (A.D.T.); 2 Fundeni Clinical Institute, 022328 Bucharest, Romania; 3 Department of Gastroenterology and Hepatology, Elias Emergency University Hospital, 011461 Bucharest, Romania

**Keywords:** gastrointestinal (GI) aGVHD, gastrointestinal ultrasound (GIUS), contrast-enhanced ultrasound (CEUS)

## Abstract

Acute gastrointestinal graft-versus-host disease (GI aGVHD) is a significant and life-threatening complication in patients undergoing allogeneic stem cell transplantation (allo-SCT). Early diagnosis of GI aGVHD is crucial for improving patient outcomes, but it remains a challenge due to the condition’s nonspecific symptoms and the reliance on invasive diagnostic methods, such as biopsies and endoscopic procedures. In recent years, interest in non-invasive diagnostic techniques for graft-versus-host disease has increased, with contrast-enhanced ultrasound (CEUS) being one of them. For this reason, we aimed to examine the potential of ultrasound as a non-invasive, safe, and cost-effective alternative for the early detection and monitoring of GI aGVHD in this review. Our narrative review aims to describe the use of multimodal US that includes conventional US (B-mode and Doppler US) and advanced ultrasound techniques such as CEUS and CRTE for the non-invasive diagnosis of GI GVHD. We browsed several databases, including PubMed, Scopus, Web of Science, and Google Scholar. The search spanned 2000 to the present, focusing on articles written in English that reviewed the use of these imaging techniques in the context of GI GVHD. Following our research, we noticed that CEUS offers several advantages, including the real-time visualization of the gastrointestinal wall, assessment of blood flow, and detailed microvascular analysis—all achieved without the use of ionizing radiation. This feature makes CEUS an appealing option for repeated assessments, which are often necessary in monitoring the progression of GI aGVHD. When used in conjunction with conventional gastrointestinal ultrasound (GIUS), CEUS provides a more comprehensive view of the structural and functional changes occurring in the GI tract, potentially enhancing diagnostic accuracy and allowing for earlier intervention. In comparison to traditional diagnostic methods like tissue biopsy or CT scans, CEUS is less invasive, quicker to perform, and better tolerated by patients, especially those in fragile health following allo-SCT. Its non-invasive nature and ability to provide immediate imaging results make it a valuable tool for clinicians, particularly in settings where minimizing patient discomfort and risk is paramount. However, despite these advantages, there are still gaps in the literature regarding CEUS’s full diagnostic accuracy for GI aGVHD. Further research, including larger clinical trials and comparative studies, is needed to validate CEUS’s role in routine clinical practice and to establish standardized protocols for its use. Nonetheless, CEUS shows considerable potential to transform the diagnostic approach to GI aGVHD by improving early detection, reducing the need for invasive procedures, and ultimately enhancing treatment outcomes for affected patients.

## 1. Introduction

Allogeneic stem cell transplantation (allo-SCT) is a potentially curative therapy for different life-threatening hematologic disorders. One of the most frequent complications post-allo-SCT is acute graft-versus-host disease (aGVHD), a complex immune-mediated process occurring in 20% to 50% of allo-SCT patients. It occurs when the donor’s immune cells (graft) recognize the recipient’s body (host) as foreign and mount an immune response against the host tissues. This immune-mediated attack can affect multiple organs, most commonly the skin, liver, and gastrointestinal (GI) tract. GVHD is classified into two types, acute and chronic, based on the timing of onset and clinical features [1,2].

The pathophysiology of acute GVHD (aGVHD) is thought to follow a three-phase process. It begins with tissue damage caused by the conditioning regimen, which activates host antigen-presenting cells through pathogen-associated molecular patterns (PAMPs) and damage-associated molecular patterns (DAMPs). This leads to the activation and proliferation of donor T cells (afferent phase). In the final effector phase, cytotoxic cells cause tissue damage by releasing inflammatory cytokines, such as interleukin-1 (IL-1) and tumor necrosis factor-alpha (TNF-α), resulting in tissue necrosis. The development of aGVHD is also influenced by immune-regulating cells, including T-regulatory cells (Tregs), type 1 regulatory T cells (Tr1 cells), invariant natural killer T (NKT) cells, and myeloid-derived suppressor cells (MDSCs), which modulate the immune response [1].

Since the introduction of post-transplant cyclophosphamide (PTCy) as graft-versus-host disease (GVHD) prophylaxis, the risk of developing this transplant-related complication has been diminished for patients who receive a transplant from an HLA-mismatched donor [3,4]. The gastrointestinal (GI) tract is among the most common sites affected by aGVHD, and grades III to IV are associated with increased morbidity and mortality [1].

Specific histopathological changes are essential for diagnosing and differentiating GI GVHD from other GI conditions. Histopathological findings for GI GVHD include cryptic apoptosis, epithelial cell damage, lamina propria inflammation, crypt destruction and loss, Paneth cell depletion, mucosal atrophy, and vascular changes [5,6,7,8,9,10].

Classical histopathological diagnostic criteria for GI GVHD are defined through the Lerner score, which is based on the presence of apoptosis of crypt or gland epithelium, crypt destruction, and mucosal denudation [6]. Lesions specific to GVHD might not be visible under endoscopy or might have overlapping macroscopic aspects with other conditions. Histopathological assessment, essential for an accurate diagnosis, is considered the gold standard for confirming GVHD diagnosis [6].

Acute gastrointestinal graft-versus-host disease (GI aGVHD) can involve the upper and lower GI tract, most frequently simultaneously, but these can also occur independently. The clinical picture of lower GI tract involvement is dominated by secretory voluminous diarrhea with abdominal pain and fever, but in severe cases, patients can also experience ileus and hematochezia. The symptoms are not specific to this complication and can occur in other conditions, such as infections and toxicities related to the administered therapies [4,11]. The higher the grade of aGVHD experienced by the patient, the shorter the overall survival registered at one year: 70% with grade II aGVHD and 40% with grades III–IV aGVHD [12]. Early diagnosis of GI aGVHD is difficult because of the nonspecific nature of the associated symptoms and numerous differential diagnoses. The current gold standard for diagnosing GI aGVHD is based on the histopathological analysis of colorectal or gastric mucosa biopsy obtained through lower or upper GI tract endoscopy. Although they are minimally invasive, the procedures may carry a higher risk for complications such as bleeding or perforation in patients with severe thrombocytopenia. Moreover, they are time-consuming and necessitate special preparation (ileo-colonoscopy, for example), thus delaying diagnosis and treatment [13]. On the other hand, the most frequently affected segment of the GI tract is the ileum, followed by the stomach, jejunum, duodenum, and rectum. This means that there may be situations where the results may appear as false negatives if the biopsies are not performed from the affected areas.

Unfortunately, there are no validated non-invasive diagnosis tests for the early detection of GI aGVHD. For nearly 20 years, several studies have concentrated on looking for GVHD-related imaging to help the diagnosis, early prediction, and treatment outcome, such as contrast-enhanced computed tomography (CT-scan), magnetic resonance enterography (MRE), PET-CT, conventional ultrasound (US), and the recently added contrast-enhanced ultrasonography (CEUS) and compound real-time elastography (CRTE).

Thus, more studies are increasingly focusing on assessing the relevance and reliability of the US and CEUS as diagnosing procedures, which may become helpful tools for diagnosing GI aGVHD in the future [14]. Our review aims to describe the use of multimodal ultrasound, which includes conventional ultrasound (B-mode and Doppler US) and advanced ultrasound techniques such as CEUS and CRTE, for the non-invasive diagnosis of GI GVHD. Table 1 highlights the main advantages of using CEUS in diagnosing GI GVHD.

## 2. Materials and Methods

**Figure 1:**
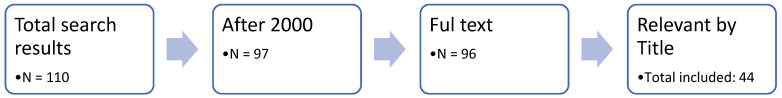
Review flowchart.

We performed a narrative review to describe the diagnostic potential of GI aGVHD using gastrointestinal ultrasonography (GIUS) and contrast-enhanced ultrasound (CEUS). We browsed several databases, including PubMed, Scopus, Web of Science, and Google Scholar. The search spanned from 2000 to the present day, focusing on articles written in English that analyzed the use of these imaging techniques in the context of GI GVHD. To ensure our assessment is relevant, we established criteria that guided our selection process. Among the inclusion criteria were patients diagnosed with GVHD with gastrointestinal involvement after HSC allotransplantation, and at least one of the ultrasonographic techniques, such as B-mode ultrasonography, contrast-enhanced ultrasonography, elastography, or Doppler ultrasonography, must have been used for the diagnosis. We also monitored the extent to which patients diagnosed with GI GVHD and underwent one of these techniques had a definitive diagnosis through histopathological examination from a digestive biopsy. Studies were chosen based on their direct relevance to GI GVHD and the technique used; only abstracts were excluded. Figure 1 shows the review flowchart and the number of articles involved.

## 3. Results

### 3.1. General Information

The US is a non-invasive, non-irradiating, and portable technique that quickly provides essential information for doctors, sometimes right at the patient’s bedside. It provides useful information about parenchymal organs and cavitary organs such as the gastrointestinal tract (GIUS). GIUS is used to evaluate and describe the five layers of the wall of the gastrointestinal tract, which can be visualized almost completely through this procedure. GIUS is usually used to assess inflammation in the gastrointestinal tract. B-mode US gives us anatomical information about the structure and integrity of the intestinal wall and its layers, while also allowing for measurements. Dynamic data on normal or pathological intestinal motility can be obtained in B-mode. Important information on the macro- and microvascularity of the intestinal wall is provided by color Doppler, power and pulse examination, and CEUS [14,15]. CRTE can also demonstrate intestinal wall stiffness with fibrotic changes, but its efficacy is still under study. However, differences from normal bowel are small and may not be clinically significant [16]. The method accurately detects severe intestinal fibrosis in patients with Crohn’s disease [17]. Qualitative, color-coded elastography appears to be better able to demonstrate signs of increased stiffness or edema and differentiates them from the normal intestine [18].

In 2019, EFSUMB published the first Guidelines for GIUS use in clinical practice, which contain the method’s indications, clinical recommendations, and US examination technique [19].

The main indications for GIUS are detecting activity and complications of inflammatory bowel diseases (IBDs) [20]. Monitoring the treatment efficiency in IBDs is essential to improving and adapting the therapeutic strategy. Thus, the US has become increasingly important in managing IBD, as multiple US indices aid in the evaluation [15,21,22].

CEUS is frequently used in IBD to demonstrate wall perfusion. CEUS is also used for the early evaluation of the response to therapy, quantifying the intensity of the wall perfusion [23]. Until now, the extravasation of contrast agents (CAs) into the intestinal lumen has not been proven in IBD.

### 3.2. Comparative Analysis of Imaging Techniques

The GVHD diagnosis is based on clinical, biological, and histopathological data. Since the clinical manifestations of GVHD are relatively nonspecific and can overlap with other processes such as infections and drug toxicity, the histopathologic evaluation of the gastric and colorectal mucosa biopsy is still necessary in many cases. It remains the gold standard for GI GVHD diagnosis [24]. Generally, endoscopy and colonoscopy are time-consuming, and due to severe secondary thrombocytopenia in transplanted patients, there is a higher risk of complications (like severe bleeding or perforation) than in other categories.

Also, if the biopsy is collected from an unaffected segment of the digestive tract, there is a risk of false-negative results.

For these reasons, multiple imaging techniques, better tolerated by patients but more or less specific for GVHD, have been tried in the last 20 years. The goal was to identify an accessible and easily used imaging technique with the highest possible sensitivity and specificity in the positive and differential diagnosis of GVHD to replace histopathology examination. These imaging techniques are mainly focused on demonstrating the inflammation of the intestinal wall and its location and identifying the presence of local or distant complications. For many of these procedures, contrast agents are necessary.

CT scan and MRE are the most frequent techniques used for GVHD diagnosis. Although CT scan and MRE are non-invasive procedures, they require intravenous and/or peroral contrast agents, which may affect the patient’s renal function and pose the risk of allergies and anaphylactic shock, in addition to the ionizing radiation effects of the CT examination. In clinical practice, patients after allo-SCT receive calcineurin inhibitor treatment as GVHD prophylaxis [25]. Calcineurin inhibitors are known to be nephrotoxic. For this reason, in patients with renal toxicity due to calcineurin inhibitors, the contrast agents for CT-scan or MRE cannot be administrated. Moreover, in some cases, the results of these investigations are inconclusive, unspecific, or cannot accurately grade the severity of the underlying disease [14].

In the case of aGVHD, studies have reported similar CT scan abnormalities with different patterns of lesion distribution in the GI tract according to the time of evaluation, severity grade, and whether the patients were dynamically scanned or not. The suggestive CT-scan findings were defined as bowel wall thickening associated with abnormal mucosal enhancement, bowel dilatation (small intestinal diameter > 2.5–3 cm and colon diameter > 8 cm), double-halo sign, and bowel loops with fluid accumulation. The involvement pattern of GI aGVHD could be used as a tool for management guidance and prognostication. These features need to be continuously monitored and bring a high radiation burden for this category of patients [2,26]. Even though mainly nonspecific, the radiological findings can delineate the aGVHD diagnosis from differential diagnoses. Firstly, *Clostridioides difficile* infection affects only the large intestine (*Cl. difficile* colitis) and produces severe wall thickening. Secondly, neutropenic enterocolitis (typhlitis) typically presents with both small and large intestine involvement, a more uniform distribution, more pronounced bowel wall thickening, and more frequently affects the caecum and the right colon [13,26,27].

Derlin et al. reported on the diagnostic efficacy of MRE in patients with active gastrointestinal aGVHD. The results reported an overall sensitivity of 81.5%, a low specificity of 35.7%, a positive predictive value of 71%, and a lower negative predictive value of 50%. In some cases, the utility of this method was evident with false-negative results for low-grade as well as for some high-grade aGVHD patients, implying that other tools are needed for an accurate diagnosis. The advantages of MRE consist of the absence of ionizing radiation (repetitive measurements are suitable), useful for aGVHD identification in patients at high risk for endoscopy-related complications, and the safety of gadolinium-based contrast for renal function. Some limitations can be defined as it cannot be performed in critically ill patients, it requires a longer-time to acquire in comparison to CT scan [28].

A comprehensive review critically examined the use of CT scans, MRI, and the US in GVHD diagnosis, highlighting their nonspecificity, associated risks (nephrotoxicity from CT scan, limited accessibility to MRI), and the lack of further development or recommendations for these methods [27]. Distinct from the aforementioned methods, CEUS, PET-CT, endoscope-based confocal laser endomicroscopy (eCLE), and probe-based confocal laser endomicroscopy (pCLE) are considered to be specific in investigating acute GI GVHD [27]. PET-CT, particularly using the radiotracer [¹⁸F] FDG (fluorodeoxyglucose), has shown potential in diagnosing GVHD, especially in the GI tract. However, the increased FDG uptake seen on PET-CT typically detects areas of increased metabolic activity, suggestive of inflammation and metabolic activity, which can occur in various conditions, including infections, malignancies, and other inflammatory diseases like Crohn’s disease, not only in GVHD. While PET-CT is a useful tool, it lacks the specificity to diagnose GVHD conclusively [29,30].

Studies have shown that PET-CT’s sensitivity for detecting gastrointestinal GVHD is relatively high, but false-positive results and overlaps with other inflammatory conditions limit its specificity. For example, one recent study (2023) reported that [¹⁸F] FDG PET/CT had a sensitivity of about 70% but lower specificity (around 57–76%) when distinguishing between GVHD and other conditions [29].

Therefore, while PET-CT may help map disease distribution and activity, it is not specific to GVHD and cannot be used as a standalone diagnostic tool [29,30]. On the other hand, PET-CT has the disadvantages of high cost and radiation exposure [27].

Standard ultrasound in association with echo-endoscopy was another technique that demonstrated its effectiveness, including rare and difficult-to-diagnose gastrointestinal diseases [31]. However, echo-endoscopy remains an invasive procedure, sometimes with much higher risks in thrombopenic patients after HSC allotransplantation, and there is a lack of studies in this direction.

pCLE and eCLE are minimally invasive real-time imaging techniques allowing for the in vivo microscopic examination of the gastrointestinal mucosa. Both techniques have shown results that closely correlate with histological diagnosis in GI GVHD. These imaging technologies offer a real-time, high-resolution visualization of the gastrointestinal mucosa, allowing clinicians to detect characteristic features of GVHD, such as crypt apoptosis, mucosal damage, and inflammation.

Studies have demonstrated that both methods can help guide biopsy and increase diagnostic accuracy, potentially reducing the need for repeated biopsies [32]. However, eCLE and pCLE are invasive and require specialized equipment and training, and are thus currently limited to research settings [27].

### 3.3. GIUS in Acute GVHD

#### 3.3.1. B-Mode US

Despite its lack of specificity in GI GVHD diagnosis, there are multiple studies that analyzed the contribution of multimodal US in GI GVHD diagnosis and prognosis. US is preferred due to its previously mentioned advantages, and the technical progress of the last two decades turned it into a first-line imaging technique in patients with aGVHD.

Throughout the studies, the abdomen was scanned using various convex multi-frequency transducers aiming to detect suspicious bowel loops. Suspected areas were further investigated by a high-resolution linear multi-frequency transducer, which can provide more detailed anatomic information.

The first studies analyzed the effectiveness of B-mode and Doppler US in acute GVHD severity assessment, focusing on bowel wall thickness, hypervascularization, and other key parameters affecting the GI tract. These studies suggested that the bowel wall thickening mainly resulted from a thickened submucosa, and the ileocecal region was the main location for the manifestation of gut GVHD, which is significant for diagnosis [33]. Because most of the important differential diagnoses [infections, e.g., pseudomembranous colitis or cytomegalovirus (CMV) colitis] have different manifestation patterns, the authors suggest that the characteristic sonographic finding of a thickened bowel wall in the ileocecal region in symptomatic bone marrow-transplanted patients might help to differentiate between infections and aGVHD of the bowel [33]. The same studies described in high-grade acute bowel GVHD patients on small-intestine-affected regions increased motility and a reduction in Kerckring’s plicae circulares with a loss of their uniformity as an ultrasonographic sign of secretory diarrhea. In contrast, in patients with a low-grade intestinal aGVHD, without clinical evidence of GI aGVHD, thickening of the bowel wall could be detected, but no sonographic signs of secretory diarrhea were identified. This indicates that B-mode US may detect early-stage aGVHD before developing clinical symptoms [33].

Recent studies also used conventional US to try to systematize its use in the detection, assessment, and grading of the severity of acute GVHD [34]. The main common targets for the US evaluation of the bowel were the wall thickness, further investigated if enlarged, and hypervascularization. Other points of interest that were closely monitored were the different bowel wall layers, degree of dilation and motility of bowel loops, presence of haustrations, presence of free fluid, and bowel content [27,35,36]. A high-resolution linear multi-frequency transducer further investigated areas that were found to present either wall edema or a wall thickness of over 3–4 mm. In addition, color-coded CE was performed to identify loops with bowel wall edema.

Notwithstanding that the caecum and terminal ileum are the most frequently affected areas, the large bowel was assessed in its entirety [34,37].

The most common finding in patients histologically confirmed with GVHD was the thickening of the ileum wall, followed by the stomach, jejunum, duodenum, and rectum walls. Thus, bowel wall thickness (BWT) was defined as abnormal if over 3 mm in the large bowel and over 2 mm in the duodenum and small bowel. This was used in correlation with critical hypervascularization of the mural and mesenteric vessels on CCDU and prolonged contrast enhancement on CEUS as criteria for an inflammatory reaction. Additionally, a thickness greater than 5 mm in the stomach and rectum, as well as intestinal dilation (defined as a diameter greater than 18 mm when the intestine was filled with fluid), was also considered relevant [33,34,38].

B-mode US can also provide other information, such as the presence and amount of free fluid in the abdominal cavity, mesenteric fat tissue’s echogenicity, or lymph nodes’ presence and size.

Correlating B-mode US findings with the severity of aGVHD, studies demonstrated that the degree of the wall thickening and dilation of the GI tract, as well as the number of affected segments and presence of ascites, did not correlate with the clinical stage of GVHD. On the other hand, the thickness of the internal low echoic layer (submucosa), the echogenicity of mesenteric fat tissue, and the intensity of Doppler signaling were significantly correlated with the clinical stage of GVHD [33].

#### 3.3.2. Doppler Ultrasonography

In aGVHD, as in IBD, color-coded Doppler ultrasonography (CCDU) and power Doppler (PD) examination are used in all studies to demonstrate the assessment of arterial perfusion of the bowel wall that is usually increased. This is a typical but unspecific sign of an inflammatory bowel process.

We must mention here a study published in 2001 on a small number of patients in which the velocities in the superior mesenteric artery (SMA) were also evaluated [33].

This study showed that normal or increased perfusion in the intestinal wall was identified in eight out of twelve patients (67%), while in four patients (33%), CCDU demonstrated reduced blood flow, highly suggestive of ischemia. No blood flow was detectable on CCDU or PD in the thickened bowel wall.

In these patients, a reduced blood flow in the SMA (<110 cm/s) with a modified, ischemic Doppler curve (low-amplitude band of systolic flow, without any diastolic flow) was detected. The results of CCDU correlated with the endoscopic appearance and histopathology data in which an ischemic colon wall was observed as proof of bowel wall ischemia, with no bleeding occurring after the biopsy.

These results correlated with a lack of response or only an initial response to immunosuppressive therapy. All four patients died due to GI aGVHD despite the escalation of GvHD therapy, while the other patients had favorable evolution under therapy.

The authors concluded that because only patients with reduced bowel circulation died as a result of GI aGVHD, this pattern defines an advanced stage with irreversible damage, suggesting the assessment of arterial blood flow in the SMA and bowel wall as a prognostic marker in aGVHD [33].

These data correlate with another study suggesting that the presence of mesenteric edema and the number of involved loops shown on the CT scan enabled the researchers to predict the development of GVHD with a sensitivity of 75% and specificity of 95% [11,39].

Table 2 summarizes the results obtained from studies depending on the technique used in patients with GI GVHD.

#### 3.3.3. Strain Elastography

Elastography measures tissue stiffness, which can be affected by fibrosis, inflammation, and other pathological changes. Studies demonstrated its utility in chronic liver GVHD, while its role in gastrointestinal GVHD is still emerging [42]. We found two studies that demonstrated the effectiveness of strain elastography in the diagnosis and severity grading of acute GI GVHD [14,34]. In these studies, strain elastography was performed in addition to conventional ultrasound (B-mode and Doppler US) and CEUS on prior suspicious intestinal locations to increase ultrasonographic diagnosis accuracy of aGVHD. Strain elastography was realized using the transducer to apply repetitive, minimal pressure to the tissues. Depending on their elasticity, the tissues were more or less deformed. The results are presented as a color-coded map superimposed on the B-mode image. The coding is conventional, established by the manufacturer of the ultrasound machine. Usually, the map of strain uses a scale from red (high strain, soft) to blue (low strain, hard). A stiffer area would be less deformed than an elastic one; therefore, fibrosis of the bowel wall would appear blue due to increased stiffness, while edema and inflammation would appear red due to less stiff tissue. Thus, strain elastography can clearly separate bowel loops with acute edema, as in aGVHD, from those with chronic changes in the intestinal wall.

Both studies demonstrated that elastography performed in association with conventional US (B-mode and Doppler US) and CEUS increases the accuracy of US diagnosis of aGVHD [14,34].

#### 3.3.4. CEUS in Acute GVHD

Only a few studies have been published about the contribution of CEUS in the diagnosis and monitoring of aGVHD [40,41,43]. CEUS is an imaging technique that enhances conventional US by using CA, which allows for a real-time visualization of the microvascularization of the tissues and organs. These CAs are composed of much smaller microbubbles than red blood cells injected into the bloodstream, which allows them to flow through the smallest blood vessels (capillaries). When ultrasound waves are transmitted into the body, these microbubbles produce strong echoes, creating more detailed images than the conventional US can provide. The most used CAs worldwide are based on sulfur hexafluoride microbubbles, an inert gas wrapped in a phospholipid membrane. This CA is strictly intravascular, with a very good safety profile and a low incidence of side effects [44,45,46,47,48,49].

Studies have shown that the use of sulfur hexafluoride in the pediatric population is safe; so, in 2017, the FDA approved its use in intravenous administration in children. The safety of the method and the lack of irradiation, the lack of renal, hepatic, or cardiac toxicity, regardless of the doses used, and the lack of damage to thyroid function have made CEUS an imaging technique to be considered in patients with bone marrow transplantation [50,51,52,53,54,55].

CEUS has been used to assess aGVHD with varying degrees of success. CEUS allows for gastrointestinal tract microvasculature and perfusion mapping following intravenous injection, providing anatomic and physiologic information about intestinal GVHD [56]. Initial studies showed that the penetration of the intravenously applied microbubbles in the bowel lumen was a specific sign of acute GVHD, as the patients without GVHD showed no transmural bubble penetration (including patients with viral or bacterial infections of the GI tract) [37,56]. But another study also found the passage of microbubbles from the damaged mucosa into the lumen in patients with neutropenic enterocolitis and considered that the CAs can pass from the bowel wall into the intestinal lumen when there is a damaged mucosal barrier, independent of the cause. However, the studies were conducted on a few patients, so further studies were needed.

CEUS detected microvascularization changes in the bowel and was deemed useful for monitoring therapy in a prospective study that included 83 patients admitted for allogeneic BMT. Only 14 patients developed biopsy-proven intestinal GVHD, while 16 other patients had biopsy-proven stomach GVHD without intestinal symptoms, and 4 other patients had neutropenic enterocolitis [40]. Time–intensity curves were generated for all patients, showing rapid enhancement peaks (corresponding to the microvascularization of the bowel wall) and a prolonged mean transit time. Patients with a complete response to therapy showed normalization of standard US and CEUS, similar to healthy volunteers and without GVHD patients. Furthermore, in steroid-refractory patients, CEUS became normal only in patients who achieved complete remission after salvage therapy, while it remained unchanged in patients who died of treatment-related complications.

Another prospective study showed that a composite score including morphological and vascular changes using B-mode and color-coded Doppler sonography, changes in mural stiffness using compound elastography, and dynamic microvascularization using contrast-enhanced ultrasound had a 97.6% sensitivity and 94.4% specificity for GI GVHD when compared to asymptomatic allo-SCT recipient controls [34]. As in previous reports of more traditional diagnostic approaches, there was a lower specificity (39.6%) for diagnosing severe stages of aGVHD. By developing a scoring system using bowel wall thickness, free fluid in the abdomen, microbubble penetration, Color-coded Doppler sonography, and elastography, they were able to increase diagnostic accuracy and ability to differentiate between low- and high-severity grades, raising specificity for high-grade GVHD diagnosis up to 79.2% [34].

Color-coded parametric imaging of CEUS was proven technically feasible and valuable for detecting acute GVHD in clinical practice [14]. Lower GI aGVHD was accurately diagnosed by inexperienced examiners in 89% of cases (17/19) and in 95% of cases by an experienced examiner. To establish a GI aGVHD diagnosis, the examiners used a dynamic evaluation with the following parameters: arterial hyperenhancement and microbubble penetration into the intestinal lumen. This pilot study stipulates that CEUS imaging studies could represent an advantage and future method for aGVHD diagnosis because of its reproducibility, feasibility, easy accessibility, and lower toxicity [14].

## 4. Discussions

New, non-invasive, and less-aggressive diagnostic and monitoring tools are continuously evaluated. This review shows that diagnosing GI aGVHD through less-invasive diagnostic methods is particularly interesting. Unfortunately, there are no standardized non-invasive imagistic approaches for diagnosing and monitoring GI aGVHD.

Even if CT scans and MRI have proven to have a high specificity and sensitivity for diagnosing aGVHD, these methods still pose significant disadvantages and supplementary risks for this category of patients. The ionizing radiation for CT scans presents the following main disadvantages: the long acquisition time, reduced availability for MRI imaging, and the nephrotoxicity of some contrast substances used for these techniques. As a result, the medical community continued to search for rapid, reproducible, less toxic, and more affordable diagnostic tools that could fulfill the non-invasive criterion.

The use of CEUS offers a promising, non-invasive alternative for the early diagnosis and monitoring of GI aGVHD, a severe complication following allo-SCT. Traditional diagnostic methods, such as histopathological analysis via endoscopy, are invasive and carry certain risks, particularly in patients with severe conditions like thrombocytopenia.

CEUS is currently the newest diagnostic approach under consideration for intensive research in this category of patients. CEUS provides several advantages over current diagnostic techniques. It is a safe procedure that does not involve ionizing radiation, making it a more suitable option for repeated use. Additionally, it is cost-effective, accessible, and can be performed quickly, providing clinicians with real-time visualization of the gastrointestinal tract wall, blood flow, and microvascular structures. These characteristics make it highly valuable for diagnosis and the monitoring of the response to treatment in patients with GI aGVHD.

By combining CEUS with GIUS, clinicians can obtain a more comprehensive view of the gastrointestinal wall’s anatomy, inflammation, and vascular changes. Although further validation through clinical trials is required, CEUS has the potential to become a reliable and non-invasive diagnostic tool, improving early detection and treatment outcomes for patients with GI aGVHD.

Although CEUS is recognized as a non-invasive, easy-to-use diagnostic method generally well tolerated by patients, there remain significant gaps in the current literature regarding its application in diagnosing aGVHD, particularly in gastrointestinal manifestations. One important gap is the absence of standardized protocols for its use in aGVHD diagnosis. While CEUS has demonstrated potential for identifying microvascular changes and inflammation within the gastrointestinal tract, no universally accepted guidelines outline when and how the procedure should be performed for aGVHD patients. This lack of uniformity makes comparing results across different studies and medical centers difficult, limiting its clinical adoption.

Disadvantages and limitations to this method could include inter-examiner subjectivity, lower specificity, and an inability to evaluate the upper gastrointestinal tract. Notwithstanding the multiple positive aspects of CEUS in the diagnosis of lower GI aGVHD, the limited number of patients and studies in the current literature highlight the need for continuous research in this field to prove the method’s specificity and diagnostic quality.

## 5. Conclusions

CEUS is a promising new diagnostic tool for GI aGVHD. It offers advantages such as being safe (no ionizing radiation), cost-effective, and capable of providing real-time visualization of the GI tract’s anatomy and blood flow.

Combination with GIUS enhances CEUS’s diagnostic capability by offering a more detailed view of the gastrointestinal wall, inflammation, and vascular changes, which can be particularly useful in the early detection and monitoring of GI aGVHD.

## Figures and Tables

**Table 1:** The key benefits of CEUS.

The Key Benefits of CEUS in Diagnosing GI aGVHD:
It is a safe, non-invasive technique. It avoids the use of ionizing radiation. It is cost-effective and readily accessible. It can be performed quickly and with ease. It provides real-time visualization of the GI tract wall, blood flow, and microvascular structures. It allows for monitoring the response to treatment.

**Table 2:** Results obtained from studies depending on the technique used.

		Techniques Used	GVHD-Proven Patients	Findings	Control Lot	Findings
A.G. Schreyer et al. [40]	2011	B-mode CEUS	14	- increased BW - enhancement of the bowel microcirculation - persistent microcirculation enhancement longer than 2 min	16	- thin layer of enhancement of BW
M. Zhang et al. [41]	2019	B-mode CEUS Doppler	5	- BW edema and BW thickness - increased vascularization in BW - intravenously injected microbubbles were detected in the affected BW	4	- no BW thickening - no signal increase representing microbubbles within the BW
D. Weber et al. [34]	2019	B-mode CEUS Doppler elastography	29	- penetration of microbubbles into the gut lumen - BW thickness - increase color-coded Doppler - soft BW on elastography	18	- no microbubble penetration - normal BW - normal color-code Doppler
Pausch et al. [14]	2021	B-mode CEUS elastography	21	- penetration of microbubbles into the gut lumen - BW thickness - soft BW on elastography	-	

CEUS—contrast-enhanced ultrasound; GVHD—graft-versus-host disease; BW—bowel wall.

## Data Availability

Data necessary for this review’s writing are available on PubMed, Scopus, Web of Science, and Google Scholar.
